# Anesthesia Management of Vitrectomy in a Patient with Sturge-Weber Syndrome

**Published:** 2018-01

**Authors:** Fatemeh Roodneshin, Mahtab Poor Zamany Nejat Kermany, Pooya Rostami, Hamed Tanghatari

**Affiliations:** 1 Labbafinejad Medical Center, Department of Anesthesiology, Shahid Beheshti University of Medical Sciences, Tehran, Iran,; 2 Department of Anesthesiology, Shahid Beheshti University of Medical Sciences, Tehran, Iran.

**Keywords:** Sturge-Weber syndrome, Propofol, Difficult airway

## Abstract

Sturge-Weber syndrome (SWS) is a neurocutaneous disorder, characterized by leptomeningeal angiomas involving the oral cavity, trachea, larynx, and face. Herein, we present a case of vitrectomy in a seven-year-old boy with SWS. The patient showed hemangioma on the left side of his face, as well as mental retardation and epilepsy. Preoperative examination revealed no apparent hemangioma in the oral cavity, pharynx, larynx, or trachea. However, he was predicted to have difficult airway intubation, as the oral cavity was smaller than the normal size. The minimum Mallampati score was 3–4 due to macroglossia. First, we applied awake intubation, but he failed to follow the commands. We proceeded to general anesthesia with propofol and did not use any muscle relaxants to maintain spontaneous breathing. A laryngeal mask airway was inserted to minimize any harm to possible oral angiomas. The patient was hemodynamically stable and extubated without any complications, such as bleeding or respiratory problems.

## INTRODUCTION

Sturge-Weber syndrome (SWS) is a sporadically occurring anomaly of embryonic development, associated with a somatic activating mutation in guanine nucleotide-binding protein (q) alpha-1 subunit (1). Patients with SWS need to be assessed for any possible airway or central nervous system (CNS) anomalies. Due to the high risk of bleeding in angiomatous lesions and upper airway deformities, difficult airway management has always been a matter of concern to anesthesiologists. In order to be prepared for any possible difficulties in airway management, multiple face mask sizes, endotracheal tubes, laryngoscopy blades, and handles should be available in advance. Video laryngoscopes and fiber-optic intubation may also prevent injury to any airway hemangiomas. In addition, a difficult airway trolley should be accessible for substitute oxygenation policies. Common head positioning, mask ventilation, and intubation are difficult due to the facial asymmetry of SWS patients. Therefore, it is critical to take ample time and prepare patients for optimal positioning with pillows and/or blankets if necessary. On the other hand, emotional distress with its potential hemodynamic effects can cause swelling of hemangiomas and lead to the increased risk of perioperative bleeding. The risk of bleeding can be minimized by ensuring adequate preoperative examination and selecting appropriate premedication (2).

Vascular angiomas may involve the tongue, larynx, gingiva, nose, palates, and trachea, causing challenges in laryngoscopy and intubation. Presentations vary from superficial skin involvement to extensive systemic and airway involvement (2). Since SWS is a neurocutaneous syndrome, patients may suffer from other neurological and ocular problems including glaucoma. Patients with glaucoma require smooth induction and intubation to avoid an abrupt rise in intraocular pressure.

## CASE SUMMARIES

**Figure 1.**
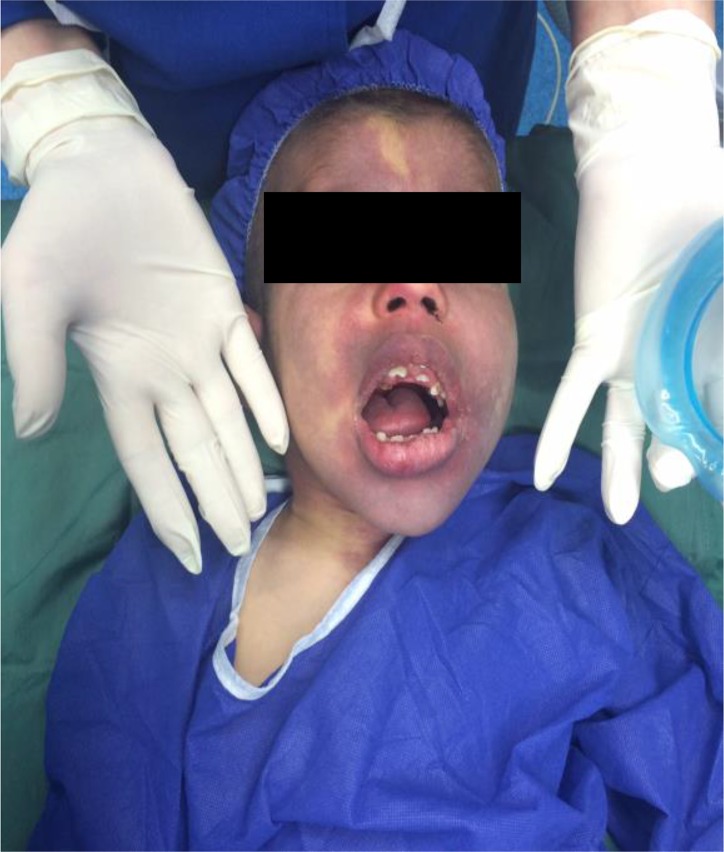
Left-sided hemangioma on the face

A seven-year-old boy with SWS, characterized by left-sided, red-purple discoloration of his face, was admitted to the ophthalmology department of Labbafinejad Hospital for vitrectomy after prolonged and unsuccessful noninvasive treatment of glaucoma of the right eye (Figure 1). Diagnosis of SWS was confirmed, based on the cutaneous port-wine stain, history of intractable seizure and glaucoma, and tram-track pattern of brain CT scan.

The patient’s weight was 15 kg, and he had undesirable management of ipsilateral glaucoma. He had a history of generalized and focal seizures, treated with phenobarbital, levetiracetam, and sodium valproate. He showed moderate mental retardation and could hardly speak. His neurology consultation indicated no risky hemangioma in the brain. Consultation with an otolaryngology specialist revealed no further elucidation of lesions using upper airway endoscopy. No other significant medical or surgical histories were existing.

**Figure 2.**
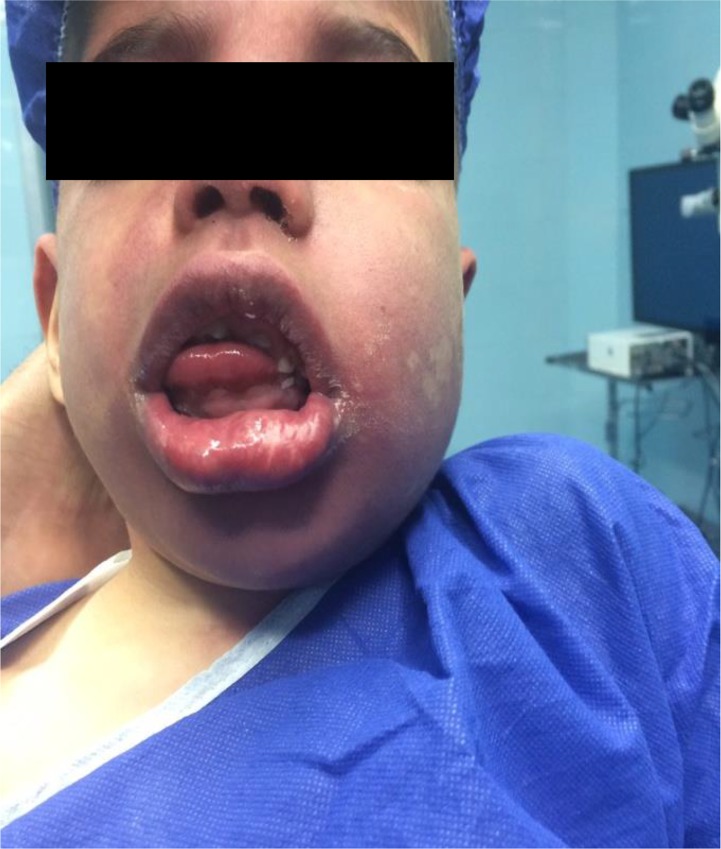
Poor dentition, gingival angiomatoid hypertrophy, large tongue

An informed consent was obtained from the parents. Upon the patient’s arrival to the operating room, a thorough and systematic physical examination was performed. The physical examination revealed a dominant left-sided hemangioma of the face, distributed in the three branches of trigeminal nerve, arm, forearm, and lower extremity. Poor dentition, gingival angiomatoid hypertrophy, large tongue, and hypertrophied tonsils were also noted (Figure 2).

Sensory and motor functions were found to be normal. The angiomatoid lesions were warm to palpation and blenching with compression. Neck extension was normal, and the mouth opening was two-finger wide. Blanching gingiva suggested the same angiomatous anomalies. The patient had slight breathing difficulties in the supine position.

The patient’s Mallampati score was III–IV due to macroglossia. Therefore, he was speculated to have difficult ventilation and intubation considering his Mallampati score of IV, Cormac III, and upper lip bite test (ULBT) II classification (Figure 1). Preparations were made for any possible airway difficulties. We used immediately-available fiber-optic laryngoscope blades (straight and curved) in different sizes, video laryngoscopy, and various sizes of laryngeal mask airways (LMAs), endotracheal tubes, and capnography for ensuring appropriate ventilation. Although the attending anesthesiologist had ample expertise in difficult airway management, a general surgeon was available for possible cricothyrotomy or tracheostomy if needed.

Due to his fragile and vulnerable gingiva, clinically obvious difficult airway management, increased intraocular pressure, preexisting shortness of breath, and oral mucosal angiomatous overgrowth, we decided to use a LMA (size=2). Routine cardiovascular and respiratory monitoring, including electrocardiography, pulse oximetry, non-invasive blood pressure (NIBP) measurements, end-tidal CO_2_ analysis, bispectral index (BIS), axillary thermometer, and an 18-gauge angiocatheter of the left hand were used.

The patient’s vital signs were as follows at baseline: blood pressure, 125/65 mmHg; pulse rate, 89/min, O_2_ saturation, 95% in room air; and body temperature, 36.7°C. He was preoxygenated with 100% O_2_ for 5 minutes in the supine position. Premedication was initiated with intravenous midazolam (1 mg) and alfentanil (500 μg). Lidocaine (20 mg) was added to minimize intubation responses. General anesthesia was induced with propofol (50 mg) and remifentanil (30 μg), respectively, while ventilation was acceptable. Following that, a well-lubricated LMA (Number 2) was successfully inserted.

A Mapleson F system with continuous positive airways pressure (CPAP) was used to assist spontaneous ventilation. The patient was ventilated properly, and the capnogram was satisfactory. Dexamethasone (2 mg) was intravenously injected before the operation. Anesthesia was maintained with intravenous propofol and remifentanil (propofol at 200 μg/kg/min and remifentanil at 1 μg/kg/min), as well as oxygen (6 L/min). The BIS index was also maintained at 40–50. The patient was hemodynamically stable during 90 minutes of operation and was successfully extubated while monitoring motor function and consciousness. He was then transferred to the recovery room with oxygen administrated trough a face mask. The patient’s status was satisfactory, and he was discharged from the hospital after three days.

In this high-risk patient, by following the mentioned systematic approach and adequate preparation, successful airway management and anesthesia were possible, without any tissue damage or other complications; in fact, there were no laryngospasms or bleeding of vulnerable angiomatous airway.

## DISCUSSION

Sturge-Weber Syndrome, also known as encephalotrigeminal angiomatosis, is a nonhereditary neurocutaneous syndrome with angiomas, which affect leptomeninges (leptomeningeal angiomas) and face skin, usually in the ophthalmic (V1) and maxillary (V2) branches of the trigeminal nerve (3, 4). These patients usually require several corrective procedures, such as dental procedures, trabeculectomy, eye examination, and surgery for convulsion (5), which generally need anesthesia. Ventilation, laryngoscopy, and intubation may be difficult because of angiomatic lesions in the airway (oral cavity or trachea). Manipulation of the oral cavity and airways may harm angiomatous vessels and cause severe bleeding (6). Therefore, anesthesiologists should be qualified experts and gently intubate patients to minimize injury. Overall, use of a well-lubricated, non-stylet LMA or a cuffed endotracheal tube is preferable.

Based on our experience with this patient, we found a proper position in the event of difficult airway management in children, which is the only method or conduit for endotracheal intubation (7). Uncontrolled epilepsy and use of multiple drugs can sometimes disturb anesthetic management. Therefore, decision-making regarding intravenous or inhalation anesthesia induction is important. During inhalation or intravenous anesthesia induction, upper airway obstruction may be observed, while intravenous induction has the advantage of speed and absence of unpleasant smell sensation. Moreover, in inhalation anesthesia, anesthesiologists should be completely aware of the progression of anesthesia induction. Any stimulation before the end of eyelash reflex may cause laryngospasm; yet, intravenous insertion can be attempted two minutes later (8).

An important and exceptional factor in propofol application is the way LMA can be implanted. In fact, propofol decreases the upper pharyngeal muscle force to ease the insertion of LMA (9). Propofol is also desirable for induction, as it is associated with reduced airway complications, such as bronchospasm and laryngospasm, and lower incidence of nausea and vomiting (with rapid emergence) (10). Propofol accompanied by airway caliber loss is reversed by CPAP. In fact, CPAP provides pneumatic support to increase the competence of pharyngeal airway, increases longitudinal pressure on the airway, and decreases the collapsibility of the upper airway (11).

One of the ideal techniques for patients with an abnormal airway anatomy and predicted poor cooperation is assisted spontaneous ventilation in general anesthesia. In difficult airway management, several possibilities of failure or loss of airway should be considered and planned in advance. On the other hand, accessibility of a skilled assistant, particularly a surgeon who is a qualified expert in pediatric bronchoscopy and tracheostomy, is a matter of concern (10).

Control of hemodynamics, intracranial pressure, and intraocular pressure stability during the procedure is also a matter of concern. In our patient, we decided to use a minimally invasive strategy to manage the airway because of the fragile condition of gingiva, besides the predicted airway management difficulties and preexisting shortness of breath following subglottic stenosis. We used a lubricated LMA with a smaller size to avoid any traumas to the oral cavity.

As mentioned earlier, considering the advantages of simultaneous propofol and LMA use, induction was initiated with propofol to reduce the incidence of laryngospasms. Lidocaine and remifentanil were also added to minimize intubation stress. LMA was inserted gently to avoid bucking, straining, and airway obstruction as far as possible. In a predicted difficult airway, access to a rapid and secure airway is preferred; therefore, careful intravenous induction without any muscle relaxants was selected in our patient. Although adequate hypnosis in combination with a muscle relaxant can reliably facilitate tracheal intubation, validation of tracheal intubation without muscle relaxants may not be clear to many specialists. However, if muscle relaxants are avoided, the potential complications of their use, misuse, and antagonism will be inhibited.

Many clinicians normally avoid neuromuscular blocking agents, except when clinically indicated. It is difficult to make any precise recommendations, as clinical opinion is often based on individual experiences, and regimens may differ among clinicians (12, 13). Moreover, use of neostigmine as a reversal agent is associated with an increase in salivation and postoperative nausea and vomiting (12), which can accordingly affect postoperative management; we decided to insert an LMA due to the discussed reasons. Neuromuscular blockade does not influence the ease or rate of success in LMA insertion (14).

Instead of inhalation anesthesia, we used propofol for maintenance via assisted spontaneous ventilation with CPAP to avoid any spasms in the upper airway. Moreover, the lower incidence of nausea and vomiting associated with propofol facilitated postoperative management. No reversal agent was needed, as we did not use any muscular blockades. With the help of an expert anesthesiologist in difficult airway management, not only intubation, but also emergence (transition from the sleep state to full consciousness), was adequately smooth. Moreover, no damage to the oral or airway angiomatous lesions (resulting in bleeding) was reported.

## CONCLUSION

Anesthetic management of patients with SWS is quite challenging due to the risk of difficult airway management, possibility of seizure provocation, and even hemorrhage. In order to reduce the risk of intra- and postoperative bleeding, it seems appropriate to avoid further airway traumas if possible and maintain a proper anesthetic depth to protect vulnerable angiomatous tissues against unpleasant effects.
